# UPF1 impacts on mTOR signaling pathway and autophagy in endometrioid endometrial carcinoma

**DOI:** 10.18632/aging.203421

**Published:** 2021-09-14

**Authors:** Minfen Zhang, Hui Chen, Ping Qin, Tonghui Cai, Lingjun Li, Ruichao Chen, Shaoyan Liu, Hui Chen, Wanrun Lin, Hao Chen, Amanda L. Strickland, Hanzhen Xiong, Qingping Jiang

**Affiliations:** 1 Department of Pathology, Third Affiliated Hospital, Guangzhou Medical University, Guangzhou 510150, China; 2 Key Laboratory of Major Obstetric Diseases of Guangdong Province, The Third Affiliated Hospital, Guangzhou Medical University, Guangzhou 510150, China; 3 Department of Pathology, First Affiliated Hospital, Changsha 410005, China; 4 Department of Pathology, University of Texas Southwestern Medical Center, Dallas, TX 75390, USA; 5 Department of Pathology, Northwestern University Feinberg School of Medicine, Chicago, IL 60611, USA

**Keywords:** endometrioid endometrial carcinoma, mTOR, UPF1, autophagy

## Abstract

Most EEC cases are associated with activities of the mTOR pathway, which regulates protein synthesis, cell growth and autophagy. While Up-Frameshift 1(UPF1) is a key protein factor in the nonsense-mediated mRNA degradation pathway (NMD), its role in carcinogenesis of EEC remains unclear. In this study, we first evaluated the expression level of UPF1 in EEC tissues and cell lines. Then, we investigated the effect of UPF1 on cellular function and mTOR signaling pathway; these effects were further validated *in vivo*. Finally, its effect on autophagy was evaluated by western blot and GFP-mRFP-LC3 staining. UPF1 expression in the EEC tissue samples was significantly higher than that of matched normal tissue samples. Overexpression of UPF1 promoted migration and invasion of EEC cells. Conversely, depletion of UPF1 suppressed migration and invasion of EEC cells. In addition, overexpression of UPF1 increased the *in vivo* growth of our EEC xenograft tumors. Finally, UPF1 increased the activity of the mTOR/P70S6K/4EBP1 signaling pathway and inhibited autophagy in EEC cells. These findings suggest that UPF1 functions as an oncogene to promote EEC carcinogenesis. Our findings propose UPF1 as a new potential therapeutic target for EEC.

## INTRODUCTION

Endometrial cancer (EC) is one of the most common malignant tumors in gynecological system. ECs often exhibit mixed features at the clinical, pathologic and molecular levels. Within this broad spectrum of malignancies, endometrioid endometrial carcinoma (EEC) is the most common histological type. Most EECs are related with the mTOR pathway, which is responsible for regulating protein synthesis and cell growth [1–3]. mTORC1 and mTORC2 are the catalytic subunits of two biochemically distinct molecular complexes of mTOR. Activation of mTORC1 can promote ribosome biogenesis, and increase translation rates and protein synthesis [4–7]. mTORC1 activates downstream factors involved in many cellular functions, the most important ones of which are 4EBP1 and p70s6k. In addition, some studies have demonstrated that mTORC1 can inhibit autophagy through direct interaction with Unc-51 Like Autophagy Activating Kinase 1 (ULK1) complex [8, 9]. Autophagy-related genes, such as BECLIN1, Atg5, and Atg7, are included in the complex mechanisms of regulating the progression of various cancers. It is widely accepted that LC3 is a marker of the autophagic membrane, and P62 is a commonly used marker for autophagy degradation [8–11].

As a protein essential for the nonsense-mediated mRNA degradation pathway(NMD), Up-frameshift 1(UPF1) selectively recognizes and degrades mRNAs containing premature termination codons (PTCs) through a complex set of NMD factors to stop their translation [12]. The role of UPF1 in tumorigenesis has been studied recently. Notably, the expression level of UPF1 was found to be lowered in hepatocellular carcinoma tissues and gastric cancer compared to normal controls [12, 13]. Inversely, UPF1 was highly expressed in glioblastoma and lung adenocarcinoma [14, 15]. In addition, mTOR also plays an important role in the regulation of autophagy. It has been shown that mTOR inhibitors such as rapamycin or amino acid deprivation can be utilized as positive controls for inducing autophagy [16–19].

However, no study has addressed the role of UPF1 in the carcinogenesis of EEC and its effect on mTOR pathway. Thus, we hypothesized that UPF1 may regulate mTOR pathway, and also influence autography. In this study, we found that UPF1 plays an oncogenic role in EEC like in glioblastoma and lung adenocarcinoma. UPF1 was highly expressed in EEC tissues and cell lines. It could activate mTOR signaling pathway, and inhibit autophagy. *In vitro*, the overexpressed UPF1 promoted migration and invasion of EEC cells. Conversely, inhibition the expression of UPF1 inhibited invasion and migration of EEC cells. *In vivo*, the upregulation of UPF1 could promote EEC growth.

## RESULTS

### UPF1 expression was significantly higher in EEC tumor tissues than in matched normal tissues

**Figure 1:**
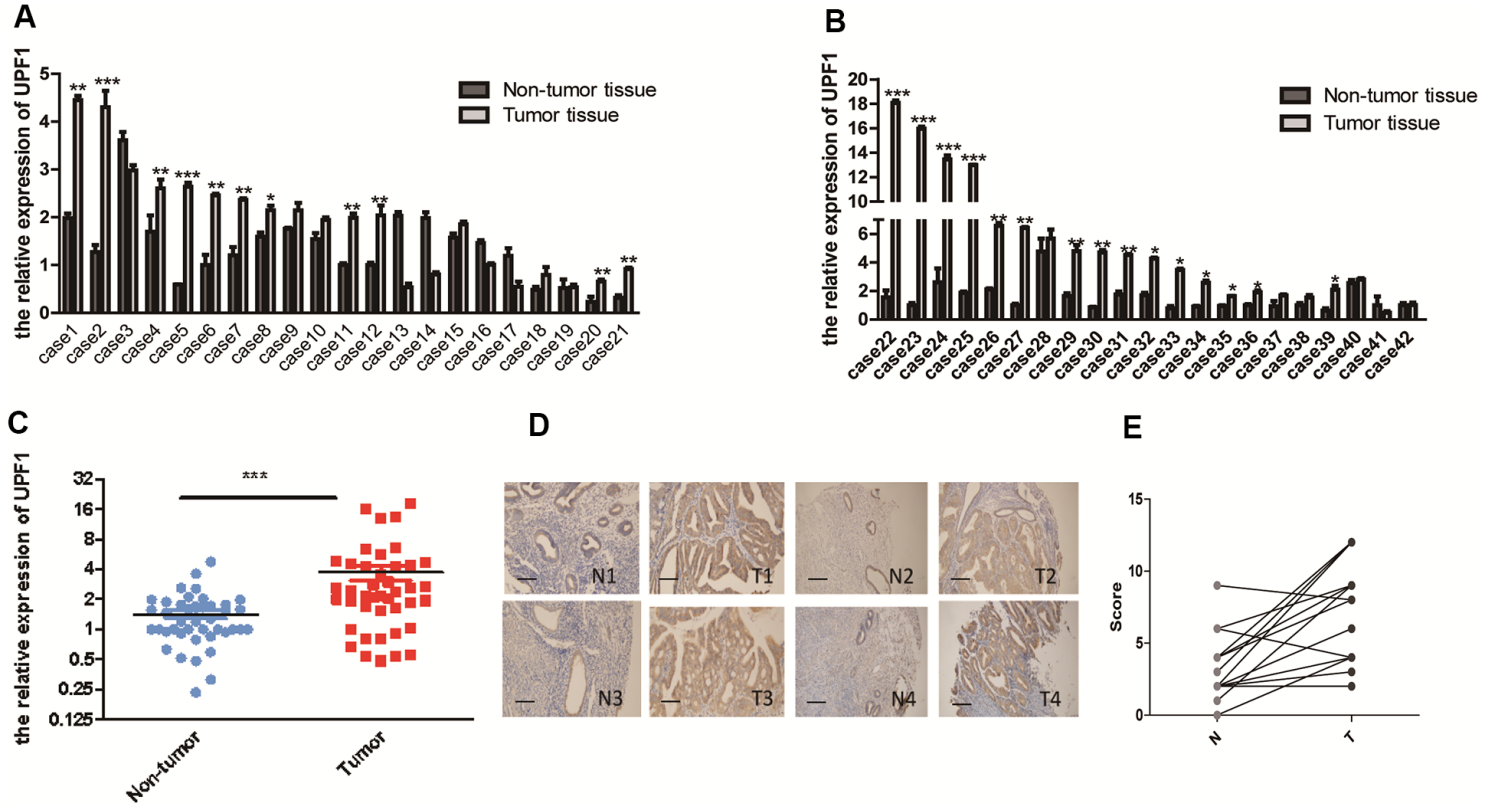
High expression of UPF1 in EEC: (**A**, **B**) 28 (66.7%) of 42 EEC patients had high expression of UPF1. In 5 of 42 cases (11.9%), the expression level was lower than normal tissues. Of the 42 cases, 9 had no significant difference with normal tissues. (**C**) Overall analysis the high expression of UPF1 in EEC is of great significance. (**D**) Immunohistochemical results showed that the positive rate of 17 (80.95%) of the 21 patients (T) was higher than that of the normal control group (N). The positive rate of 4 cases (T) (19.05%) was lower than that of the normal control group (N)(NIKON, 200X). (**E**) Scoring of immunohistochemistry. Data was represented as the mean +/- SEM.*P<0.05, **P<0.01, ***P<0.001.

To evaluate UPF1 expression in EEC, UPF1 mRNA levels from 42 paired fresh EEC tissue samples and corresponding adjacent normal endometrium were measured by real-time qPCR. UPF1 expression was higher in 28 (28/42, 66.67%) tumor samples than that in adjacent normal samples. Meanwhile, UPF1 expression in 5 (5/42, 11.90%) tumor tissues were lower than that in the adjacent tissues, and the other 9 cases (9/42, 21.43%) showed no significant difference between the tumor and the adjacent tissue (Figure 1A, 1B). Overall, the expression of UPF1 in EEC tissues was higher than that in adjacent normal tissues (Figure 1C) (p<0.001).

In addition, UPF1 protein was detected in paraffin-embedded EEC tissues of other 21 archives cases by immunohistochemistry (IHC), and the expression intensity and area of every case were evaluated under microscope. It was found that UPF1 expression in cancer tissues was higher than that in adjacent normal tissues (Figure 1E). UPF1 expression was higher in 80.95% (17/21) of EEC tissues than in adjacent normal glands. Conversely, UPF1 expression was lower than controls in only 19.05% cases (4/21) (Figure 1D). These findings suggest that UPF1 may promote EEC development.

### UPF1 promoted *in vitro* EEC cell migration and invasion

**Figure 2:**
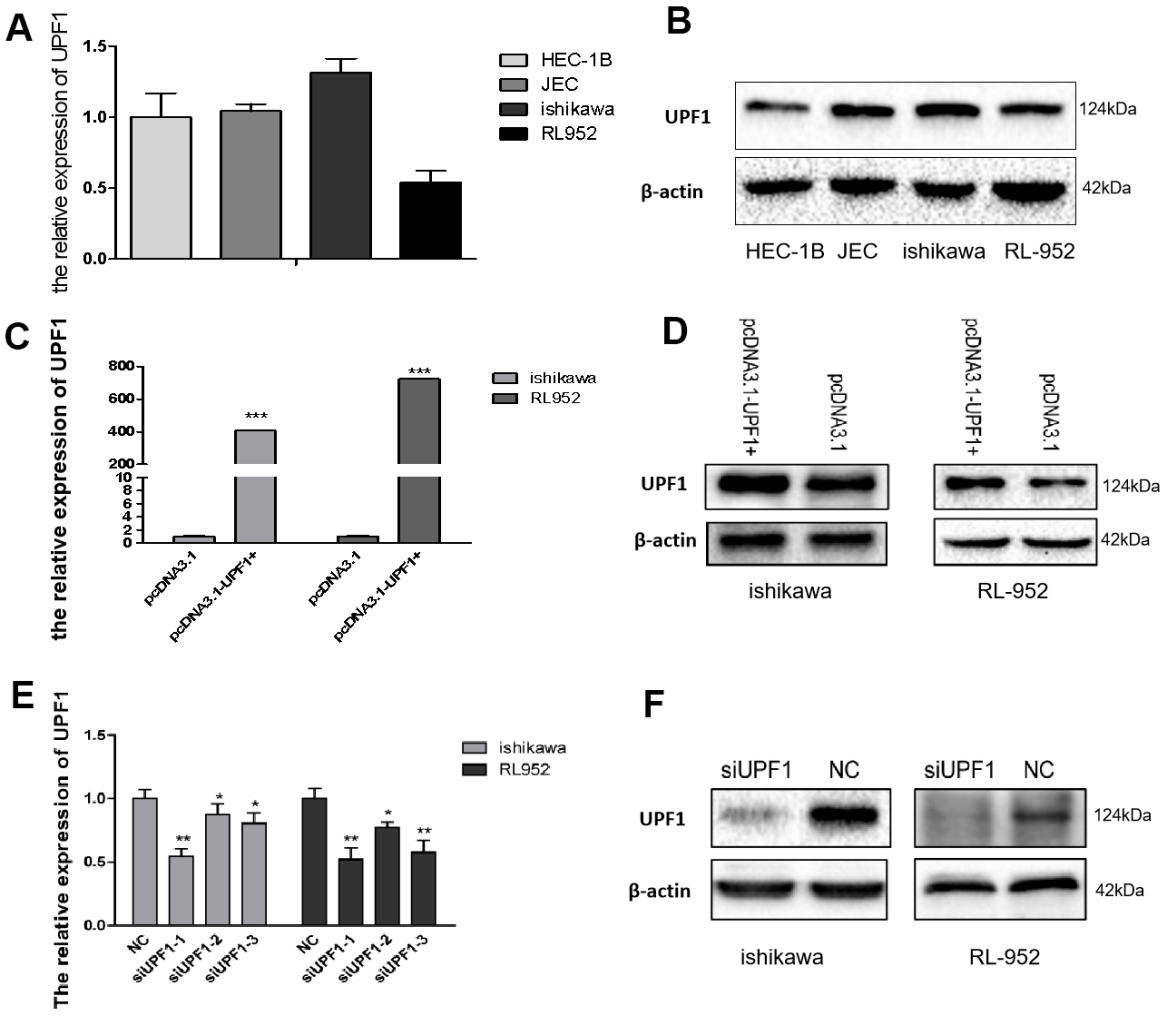
**Expression of UPF1 in EEC cells.** (**A**, **B**) UPF1 expressed level in four EEC cell lines by qRT-PCR and western blot. (**C**–**F**) UPF1 was upregulated or downregulated in Ishikawa and RL952 cells when we overexpressed UPF1(pcDNA3.1-UPF1+ and pcDNA3.1 group) or silenced UPF1(siUPF1 and NC group). siUPF1 has three fragments, we had selected the best effect in silence the expression of UPF1(siUPF1-1) to do the latter experiment.

To investigate the role of UPF1 in EEC carcinogenesis, UPF1 expression was evaluated in a panel of EEC cell lines. UPF1 expression was higher in Ishikawa cells than in the three other EEC cell lines tested (RL952, JEC, HEC-1B) (Figure 2A, 2B). UPF1 was then over-expressed in Ishikawa and RL952 cells by transfecting cells with pcDNA3.1-UPF1+ and its control pcDNA3.1 (Figure 2C, 2D). UPF1 was depleted in Ishikawa and RL952 cells by three siUPF1. One of siUPF1s which had the highest interference efficiency was chosen in follow-up experiments (Figure 2E, 2F).

**Figure 3:**
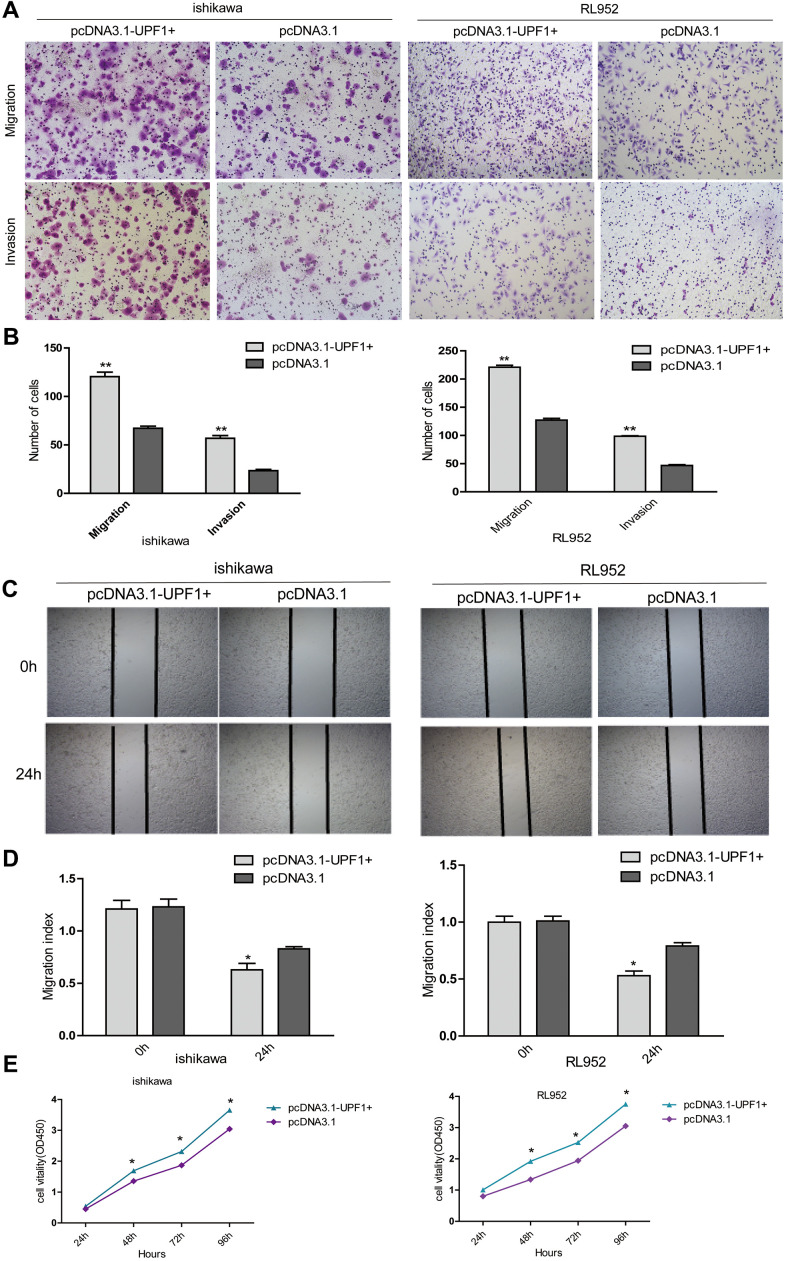
**The effect of UPF1 on cell migration, invasion and proliferation in Ishikawa and RL952 EEC cells.** (**A**, **B**) Migration and invasion assay in Ishikawa and RL952 cells that were transfected with the pcDNA3.1-UPF1+ and pcDNA3.1. Cells were evaluated at 12h after transfection(NIKON,100X). The results are shown as the mean±SEM from two independent experiments(**P<0.01). (**C**, **D**) Wound healing assay showing cell migration in Ishikawa and RL952 cells(*p<0.05). (**E**) CCK8 assay showing cell proliferation in Ishikawa and RL952 that were transfected with pcDNA3.1-UPF1+, pcDNA3.1 (*p<0.05). Data was represented as the mean +/- SEM. *P<0.05, **P<0.01, ***P<0.001.

As shown in the figures, over-expression of UPF1 promoted cell migration and invasion in both Ishikawa and RL952 cells (Figure 3A, 3B). Furthermore, the wound-healing assay showed compatible results to that of the transwell assay (Figure 3C, 3D). The results of the CCK8 assay demonstrated that increased UPF1 expression promoted cell proliferation in Ishikawa and RL952 cells *in vitro* (Figure 3E).

**Figure 4:**
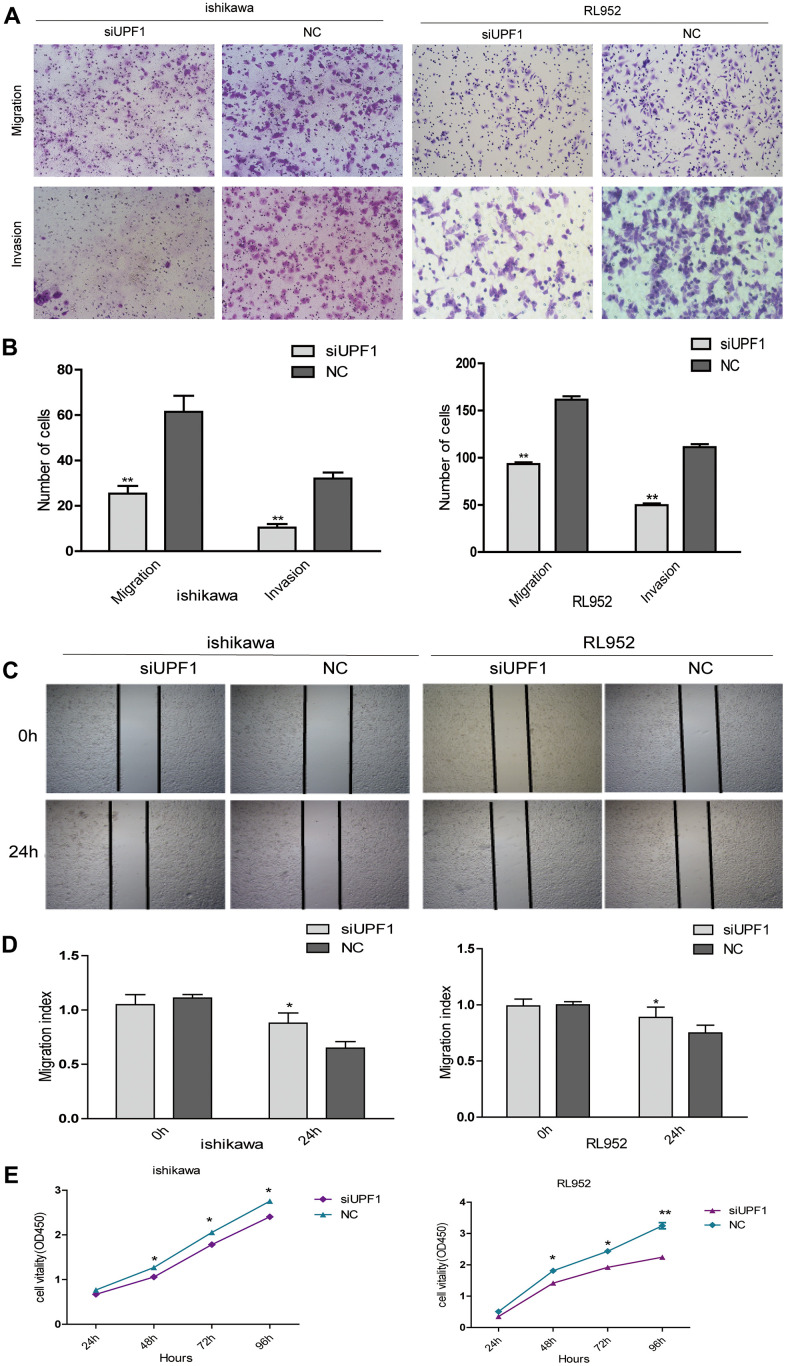
**The effect of UPF1 on cell migration, invasion and proliferation in Ishikawa and RL952 EEC cells.** (**A**, **B**) Migration and invasion assay in Ishikawa and RL952 cells that were transfected with siUPF1 and NC. Cells were evaluated at 24h after transfection(NIKON,100X). The results are shown as the mean±SEM from two independent experiments(**P<0.01). (**C**, **D**) Wound healing assay showing cell migration in Ishikawa and RL952 cells(*p<0.05). (**E**) CCK8 assay showing cell proliferation in Ishikawa and RL952 that were transfected with siUPF1 and NC(*p<0.05). Data was represented as the mean +/- SEM.*P<0.05, **P<0.01, ***P<0.001.

In order to further verify its role in EEC carcinogenesis, we silenced UPF1 in Ishikawa and RL952 cells, and showed that UPF1 depletion inhibited cellular migration and invasion (Figure 4A, 4B). Furthermore, silencing of UPF1 inhibited cellular healing ability in the wound-healing assay (Figure 4C, 4D) and inhibited cellular proliferation in the CCK8 assay (Figure 4E). Altogether, these findings indicate that UPF1 promotes EEC cell abilities of proliferation, migration and invasion.

### UPF1 could affect the mTOR signaling pathway and autophagy *in vitro*

**Figure 5:**
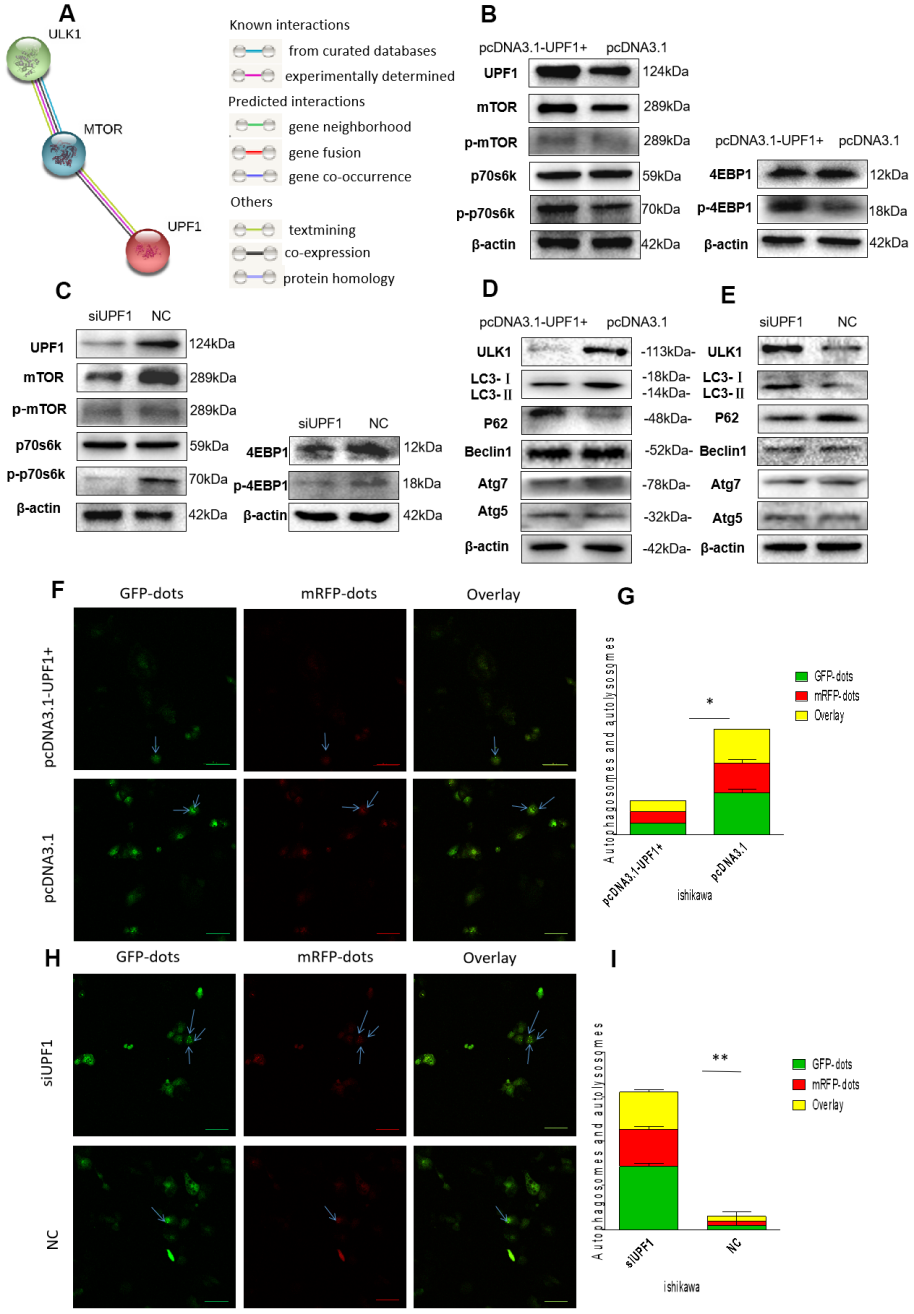
**mTOR signaling pathway and autophagy related proteins detection.** (**A**) Bioinformatics prediction (http://www.string-db.org):mTOR and UPF1 maybe co-expression and interaction. However, mTOR and ULK1 show that both of its relationship have verified. (**B**, **C**) Transfected with pcDNA3.1-UPF1+ and pcDNA3.1 or siUPF1 and NC in Ishikawa showed that UPF1 could affect mTOR and downstream’s proteins. (**D**, **E**) Overexpressed UPF1 or downregulated UPF1 could inhibit or promote autophagy. LC3I/II and p62 showed autophagy weakened or increased. (**F**–**I**) The immunofluorescence assays were performed in EEC cells that were transfected with mRFP-GFP-LC3 lentiviral in two different groups. The numbers of GFP and mRFP dots were determined by fluorescent puncta in three high-power fields. The statistical significance between different groups. Data was represented as the mean +/- SEM.*P<0.05, **P<0.01, ***P<0.001.

Nonsense-mediated mRNA degradation (NMD) inhibition induces autophagy [18]. The modest depletion of UPF1 is sufficient to inhibit NMD. mTOR signaling pathway has a central role in the regulation of autophagy. To investigate the relationship between UPF1 and mTOR signaling pathway, we first queried the protein interaction between mTOR and UPF1 by STRING database (http://www.string-db.org), which indicates that mTOR and UPF1 are co-expressed and interacted in other species and homo sapiens, though the co-expression score in homo sapiens is somewhat weak(score=0.076) (Figure 5A). Here, we demonstrated that up-regulation of UPF1 increased protein levels of mTOR and activated the downstream effectors, such as phosphorylation of p70s6k and 4EBP1 in Ishikawa cell (Figure 5B), while silencing of UPF1 conversely resulted in suppression of mTOR signaling (Figure 5C).

Moreover, overexpression of UPF1 inhibited autophagy compared with the control group. Autophagy related factors, such as ULK1 [20, 21] (the initiating factor of autophagy) and LC3I/II, were downregulated in Ishikawa cells when UPF1 was upregulated. Contrarily, when UPF1 was downregulated, LC3I/II and ULK1 were upregulated. However, beclin1, atg5 and atg7 had no changes regardless of UPF1 status (Figure 5D, 5E). It proved that UPF1 does not affect the formation of autophagy, but the degradation of autophagy.

Furthermore, we verified the effect of UPF1 on degradation of autophagy by GFP-mRFP-LC3 staining. Autophagy is a process of continuous change and development. GFP-MRFP-LC3 staining can be used to locate and detect autophagy flux, independent of changes in cellular environment pH. The acidic conditions of the lysosomal cavity make the GFP signal relatively sensitive, while the mRFP signal is much more stable. Therefore, we used a lentiviral vector carrying GFP-mRFP-LC3 to evaluate autophagic flux, autophagosome biogenesis, maturation, and lysosomal degradation. It was observed that overexpressed level of UPF1 inhibited the autophagic flux in the cytoplasm (Figure 5F, 5G). In the meantime, both the GFP/mRFP and mRFP dots were significantly reduced in the siUPF1 groups, indicating that the silencing of UPF1 increased the autophagic flux. (Figure 5H, 5I).

### UPF1 promotes tumor growth in Ishikawa cell *in vivo*

**Figure 6:**
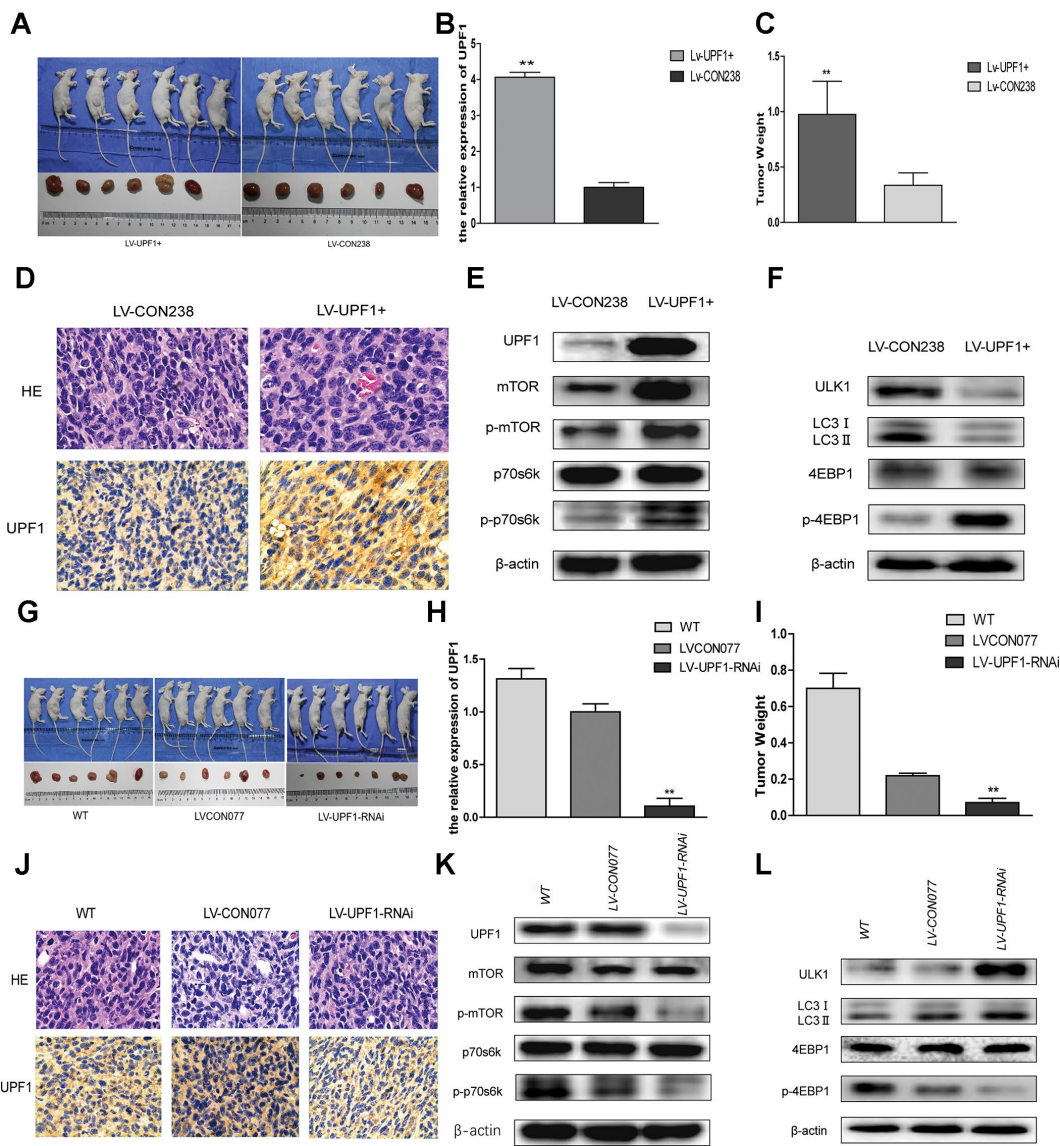
**UPF1 influenced tumor growth, mTOR signal pathway and autophagy *in vivo*.** (**A**, **G**) Photographs showing tumors that Lentivirus transfected with UPF1 stable expressed Ishikawa cell line (LV-UPF1+) and the control (LV-CON238), and that Lentivirus transfected with interfered UPF1 expressed Ishikawa cell line(LV-UPF1-RNAi), the control (LV-CON077) and wild Type group (WT). (**B**, **H**) qRT-PCR detected the relative expression of UPF1 in stable cell line. (**C**, **I**) The means tumor weights in nude mice were significant compared with the control group (**P<0.01). (**D**, **J**) H&E-stained slides (NIKON,400X) and IHC showed expression of UPF1 in tumors. (**E**, **F**, **K**, **L**) The mTOR related proteins’ and autophagy related proteins’ expression in tumors.

To further verify the above results, we constructed stable cell lines, overexpressed groups (LV-UPF1+and LV-CON238) and interference groups (LV-UPF1-RNAi and LV-CON077) using Ishikawa cell lines. *In vivo*, we transfected Ishikawa stable cell lines and wild-type Ishikawa cells to evaluate their effect on the growth of subcutaneous tumors in mice. A month later, the mice were killed and the tumor removed. The tumor volume and weight in mice with UPF1 overexpression was significantly higher than that in the control group (Figure 6A–6D). Similarly, the tumor volume and weight in mice with UPF1-inhibited cells significantly lower than that in the control and wild-type groups (Figure 6G–6J). All data suggest that UPF1 promotes tumor growth *in vivo*.

### UPF1 could affect the mTOR signaling pathway and autophagy *in vivo*

Our studies have shown that UPF1 is associated with autophagy in EEC cell lines in the previous experiments. Furtherly, we found the relationship between them *in vivo* (Figure 6E, 6F, 6K, 6L). We found that UPF1 could affect mTOR signaling pathway and autophagy.

## DISCUSSION

As a factor of NMD, UPF1 may be involved in many key cellular processes of cancer cells, such as cell differentiation, migration, invasion and cell growth. Researchers have demonstrated that UPF1 inhibits the expression of MALAT1 in gastric cancer and inhibits tumor development by targeting MALAT1. That study shows that UPF1 can inhibit the occurrence and development of tumors [13]. UPF1 is an anticancer gene for liver and stomach cancer. Conversely, other studies showed it prompts the development of glioma and lung adenocarcinoma. Briefly, it plays different roles in different tumors. However, it was not found the effect of UPF1 in EEC progress.

To investigate UPF1 expression in EEC, we examined the expression of UPF1 in EEC cases and tumor cell lines. Our results showed that UPF1 expression in tumor tissues was higher than that in normal tissues. In addition, the overexpression of UPF1 promoted the migration, invasion and proliferation of EEC cells, suggesting that UPF1 may act as an oncogene in EEC. The mechanism need to be clarified.

In glioblastoma and lung adenocarcinoma, the authors illuminated that UPF1 functioned through the interacting with some LncRNAs. However, no reference referred to which pathway was involved in after the interaction. Therefore, in our present study, we mainly focused on pathways that UPF1 may be involved.

It is known EEC development is associated with activity of mTOR pathway, which regulates protein synthesis, cell growth and autophagy. Normally, mTOR phosphorylates 4EBP1, dissociates, and activates eIF-4E to facilitate the formation of translation initiation complexes and protein synthesis [22, 23]. In addition, phosphorylation of mTOR and activation of P70S6K cause ribosome 40S small subunits to easily bind to the translation complex, improving the translation efficiency of mRNA. MTOR mainly participates in various biological functions of cells by activating downstream target protein 40S ribosomal protein S6 kinase (p70s6k), promoting protein translation, regulating protein interaction [5, 24]. It also regulates tumor autophagy process. MTORC1 is activated by nutrients and growth factors and inhibits autophagy through the phosphorylation of many autophagy-related proteins (ATGs), thus promoting the initiation of autophagy and the nucleation of autophagosomes [25]. mTORC1 also phosphorylates and prevents nuclear localization of the transcription factor EB (TFEB), a master regulator of lysosomal and autophagy gene expression [26–29].

Autophagy is conserved in the evolution from yeast to mammals, and several autophagy-related proteins (Atgs) coordinate the initiation, extension, maturation, and fusion phases of this pathway [30–32]. Critical genes for autophagy include the initiation gene ULK1 [20, 21, 33] and LC3. LC3 is divided into two forms, LC3I and LC3II, which are widely used to monitor autophagy. LC3I is cytoplasmic, and LC3II binds to cell membranes. The increase of LC3II content corresponds to the increase of autophagosome formation [34]. P62 is considered a chaperone protein during autophagosome degradation.

The relationship among UPF1, the mTOR pathway and autophagy has not been reported in endometrial cancer. In our study, we found that UPF1 can regulate the expression of mTOR, its phosphorylation and downstream proteins. We demonstrated that UPF1 up-regulates mTOR protein levels and activates downstream effector factors such as phosphorylation of p70s6k and 4EBP1 in EEC cells. In contrast, the silencing of UPF1 led to the suppression of mTOR signals in EEC cells.

Moreover, Our study shows that excessive UPF1 inhibits autophagy *in vitro* by preventing LC3I change into LC3II, and suppressing P62 degradation. Atg protein is a ubiquitin-like modified protein, which is an autophagy regulatory gene. However, no significant changes in beclin1, atg5 and atg7 proteins were detected.

The results also showed that UPF1 promotes autophagy degradation. When UPF1 was overexpressed, the expression of autophagy initiation gene ULK1 was down-regulated. Contrarily, UPF1 expression was downregulated, ULK1 expression was subsequently up-regulated. We also used GFP-mRFP-LC3 staining to prove the influence of UPF1 on autophagy flux.

In conclusion, our study has shown that UPF1 is highly expressed in EEC tissues. It can promote cell migration and invasion *in vivo*, and promote EEC growth. UPF1 plays a role of oncogene. On mechanisms, we firstly verified UPF1 can improve activity of mTOR pathway and inhibit autophagy. Further studies are needed to see if UPF1 influence autophagy totally or partly due to activating mTOR pathway.

## MATERIALS AND METHODS

### Patients and clinical samples

42 cases of fresh EEC specimens were collected, including cancer and matched normal tissue. After surgical resection, it was quickly frozen in liquid nitrogen for 5 minutes, and then stored at -80° C until RNA extraction. The specimens were gained from Third Affiliated Hospital of Guangzhou Medical University from 2014 to 2017. 21 paired paraffin-embedded specimens of EEC and adjacent benign endometrium were collected from the pathological library of Third Affiliated Hospital of Guangzhou Medical University. All patients provided informed consent, and the study was approved by the ethics committee of Third Affiliated Hospital of Guangzhou Medical University.

### Immunohistochemistry staining (IHC)

The protein expression of UPF1 (AbCAM, USA) in 21 EEC specimens was detected by immunohistochemical method. The paraffin sections were stained by DAB and then fixed with neutral adhesive after hematoxylin counterstaining. Then, a bright field microscope was used to observe and compare the expression of UPF1 in the cancerous tissues and adjacent normal tissues. The results of immunohistochemistry were reviewed by 3 experienced pathologists double-blindly. The expression grade of the protein was determined by semi-quantitative method. Specifically, the percentage and staining intensity of positive cells under the microscope are scored respectively; the percentage of positive cells: 0% is 0, 1% 25% is 1, 26%- 50% is 2, 51% -75% is 3, 76%- 100% is 4; positive staining intensity: 0 for colorless, 1 for light yellow, 2 for brown, and 3 for brown. The multiplication of the two scores is the expression grade: 0-4 for low expression and 5-12 for high expression [35].

### Cell culture

Four human EEC cell lines (RL-952, Ishikawa, HEC-1B and JEC) were ordered from ATCC and grown in RPMI 1640 (Corning, USA) or Dulbecco's modified Eagle's medium (DMEM; Corning, USA). It is supplemented with 10% fetal bovine serum (FBS; Gibco, USA) and 50 μg/ml penicillin and streptomycin. All cells were cultured at 37° C in a humid environment containing 5% CO2.

### UPF1 overexpression plasmid construction and cell transfections

The interference overexpression plasmid of UPF1 was synthesized by Sangon Biotech (Guangzhou, China) and named pcDNA3.1-UPF1+. Meanwhile, their empty vector, pcDNA3.1(+) was used as the control plasmid. 2.5ug of the UPF1 vector was transfected into EEC cells using Lipofectamine 3000 (Invitrogen, USA) according to the manufacturer's instructions.

### RNA interference and cell transfections

SiRNA against UPF1 gene and corresponding scrambled siRNA (NC) were synthesized by RiboBio (Guangzhou, China). They were transfected into EEC Cells using riboFECT™ (Ribobio, Guangzhou, China) according to the manufacturer’s instructions. The following siRNA target sequences were used: siUPF1-1, GATGCAGTTCCGCTCCATT; siUPF1-2, CCCAGACTCAAGATAACAT; siUPF1-3, GAGAATCGCCTACTTCACT. We chose one of them for relatively higher interference efficiency. The negative control siRNA sequences were as previously reported.

### Generation of stable cell line

UPF1 overexpression lentivirus (Ubi-MCS-3FLAG-SV40-EGFP-IRES-puromycin) named lv-UPF1+, and RNAi lentivirus(hU6-MCS-Ubiquitin-EGFP-IRES-puromycin) named lv-UPF1-RNAi were synthesized by GeneChem (Shanghai, China), which controls were lv-CON238 and lv-CON077 respectively. Lentivirus was transfected by the above constructs with packaging plasmids into Ishikawa cell lines. After 3 days of infection, those cells not effectively infected were killed by 5 μg/ml puromycin. The infected cells finally became a stable cell line under the maintenance of puromycin, and was verified by production of green fluorescent protein (GFP) under a fluorescence microscope.

### RNA extraction and quantitative real-time PCR (qRT-PCR)

TRIzol reagent (Takara, Japan) was used to isolate and extract total RNA from tissues. The RNA (1μg) was reversely transcribed into cDNA using a reverse transcription kit (Takara, Japan), and qRT-PCR was performed using Power SYBR green (Takara, Japan) according to the manufacturer's instructions. QRT-PCR cycling conditions were 95° C for 30s, 40 cycles at 95° C for 5s, 60° C for 30s. β-actin acts as an internal reference. UPF1 specific primer was designed (sense: 5’CTGCAACGGACGTGGAAATAC3’; reverse: 5’ACAGCCGCAGTTGTAGCA C3’); β-actin (sense: 5’CGCGAGAAGATGCCCAGATC3’; reverse: 5’TCACCGGAGTCCATCACGA3’). the ABI Step One Plus instrument (Applied Biosystems, Foster City, CA) was used to proceed qRT-PCR reactions.

### Cell proliferation assay

CCK-8 assay was used to evaluate cell proliferation of the both Ishikawa and RL-952 cell lines. After 24 hours of transfection, we planted about 8 × 10^3^ cells in a 96-well plate and cultured them for 1, 2, 3, or 4 days, and then added 10 ul of CCK-8 reagent (DOJINDO, Japan), protected from light. The cells were then placed in the incubator for 2 hours. Finally, the cells were moved into a Thermomax microplate reader to measure the OD value at 450 nm. All experiments were repeated 3 times.

### Cell migration and invasion assay

Migration chamber (8-mm pore size, Costar) and Matrigel (BD Biosciences) were utilized for *in vitro* cell migration and invasion assays. Ishikawa and RL-952 cell lines were transfected in serum-free medium for 24 hours and then inoculated in the upper chamber of transwells, while the lower chamber was filled with medium containing 10% charcoal-stripped FBS. After several hours, the cells were fixed and stained with 0.1% crystal violet. Finally, a picture of the cells on the surface of the lower chamber was taken and the cells in five random areas were count.

### Wound-healing assay

Approximately 24 hours after transfection, the cells were trypsinized and removed. Then we collected approximately 500,000 cells for each cell line, and evenly seeded the cells in 6-well culture dishes. After the cells were incubated overnight in a constant temperature incubator, a 100ul pipette tip was used to create a wound, which produced a wound field of approximately 400 nm at time zero. An inverted microscope (DMI6000B, Leica, Germany) was used to take photos of gaps immediately and at 24 hours after wounding. Statistical analysis, opening the picture with Image J software,6-8 horizontal lines were randomly drawn to calculate the distance between cells; Data processing: distance per time point - 0 hours distance = distance of cell migration. The results were expressed as a migration index. All experiments were performed in triplicate.

### Western blot analysis

Cell lysis was taken by RIPA buffer (Beyotime, China) containing PMSF. We separated aliquots of protein by 8% SDS-PAGE, and then electro-transfer to PVDF membrane (Millipore, USA). Subsequently, the membrane was blocked and the designated primary antibody was added overnight at 4° C. The next day, incubated the membrane with the secondary antibody for 2 hours. chemiluminescence assays (Pierce, USA) was used to observe signal and by a ChemiDoc-XRS + (Bio-Rad, CA), we detected and quantified protein. the band detection was within the linear range. Antibodies against UPF1 (Abcam, USA), mTOR, phospho-mTOR, P70S6K, phospho-P70S6K,4EBP1 and phospho-4EBP1 were purchased from Proteintech Technology (Wuhan, China). And autophagy related proteins, LC3I/II, p62, beclin1, atg5 and atg7 were also purchased from Proteintech Technology (Wuhan, China).

### Xenograft model in nude mice

Female BALB / c nude mice (4-5 weeks old) purchased from Southern Medical University (Guangzhou, China). Animal handling and experimental procedures approved by the Ethics Committee of Guangzhou Medical University animal experiments.

For the orthotopic models, EEC cells(1×10^7^cells for Ishikawa and its stable cell line, LV-UPF1+ and LV-CON238, LV-UPF1-RNAi and LV-CON077) in PBS were injected to form subcutaneous tumors. Each group contained 6 mice. After 3-4 weeks, the mice were euthanized. Tumors were measured by a caliper, and tumor volume was calculated as V=(smaller diameter)^2^×(larger diameter)/2. Part of the fresh tumor tissue was placed in a 4% paraformaldehyde solution, the remainder of the fresh tumor tissue was placed in -80° C refrigerator.

### GFP-mRFP-LC3 staining

The GFP-mRFP-LC3 lentivirus purchased from Shuangquan Biological Technology in Guangzhou, China. EEC cell lines cultured on coverslips were transfected with pcDNA3.1-UPF1+ or siUPF1 and its control for 24h. Then they were transfected with GFP-mRFP-LC3 lentiviral for 12h. Cells were observed under a fluorescence microscope. Green dots represented autophagosomes, and red dots represented both autophagosomes and autolysosomes. Yellow dots by the red and green channels merger represented autophagosomes, without overlapping with the red dots and green dots represented autolysosomes. GFP and mRFP number of points determined by manually counting fluorescent spots five high power field (1000 ×, Olympus).

### Statistical analyses

All data were presented as mean ± standard deviation of at least three independent experiments and P<0.05 was considered statistically significant. All tests were double-tailed and a functional analysis of variance (ANOVA) was performed. Data integration was performed using GraphPad Prism software, Windows version 5.00 (San Diego, CA, USA).

### Ethics approval

All patients provided informed consent, and the study was approved by the ethics committee of Third Affiliated Hospital of Guangzhou Medical University. All animal experiments were performed under a protocol approved by the Animal Care and Use Committee of Guangzhou Medical University. The study was conducted according to the principles outlined in the Declaration of Helsinki.
